# *BIRC5*/Survivin Expression as a Non-Invasive Biomarker of Endometriosis

**DOI:** 10.3390/diagnostics10080533

**Published:** 2020-07-30

**Authors:** Carolina Filipchiuk, Antonio Simone Laganà, Rubia Beteli, Tatiana Guida Ponce, Denise Maria Christofolini, Camila Martins Trevisan, Fernando Luiz Affonso Fonseca, Caio Parente Barbosa, Bianca Bianco

**Affiliations:** 1 Center of Natural and Human Sciences (CCNH), Universidade Federal do ABC, Santo André 09210-580, SP, Brazil; carol_f24@hotmail.com (C.F.); bianca.bianco@fmabc.br (B.B.); 2 Department of Obstetrics and Gynecology, “Filippo Del Ponte” Hospital, University of Insubria, 2100 Varese, Italy; 3 Discipline of Sexual and Reproductive Health and Populational Genetics, Department of Collective Health, Faculdade de Medicina do ABC/Centro Universitário Saúde ABC, Santo André 09210-580, Brazil; rubs.fmabc@gmail.com (R.B.); denise.christofolini@fmabc.br (D.M.C.); caio.parente@fmabc.br (C.P.B.); 4 Postgraduate Program in Health Sciences, Faculdade de Medicina do ABC/Centro Universitário Saúde ABC, Santo André 09210-580, Brazil; tatianaguidaponce@gmail.com (T.G.P.); camilatrevisan22@gmail.com (C.M.T.); 5 Discipline of Clinical Analysis, Deparment of Patology, Faculdade de Medicina do ABC/Centro Universitário Saúde ABC, Santo André 09210-580, Brazil; profferfonseca@gmail.com

**Keywords:** endometriosis, survivin, *BIRC5*, apoptosis, inhibitor of apoptosis protein

## Abstract

The etiology of endometriosis is highly complex, and although it is a benign disease, it has several biological behaviors similar to malignant lesions, including cell invasion, neo-angiogenesis, and decreased apoptosis. Survivin is a protein encoded by the *BIRC5* gene that plays a role in cell division by inhibiting apoptosis and regulating the process of mitosis in embryonic and cancer cells. Therefore, we aimed to evaluate the expression of *BIRC5* in samples of peripheral blood of women with and without endometriosis. This study comprised of 40 women with endometriosis and 10 healthy women as controls. Peripheral blood samples were collected in the three phases of the menstrual cycle (follicular, ovulatory, and luteal). The expression of the *BIRC5* gene was evaluated by RT-qPCR using the TaqMan methodology. The *BIRC5* expression was significantly higher in all phases of the menstrual cycle in women with endometriosis, regardless of the disease stage. The accuracy of *BIRC5* expression in the peripheral blood for the diagnosis endometriosis presented AUC of 0.887 (*p* < 0.001), with 97.2% of sensitivity and specificity of 65.5% considering the overall endometriosis group. Regarding the minimal/mild endometriosis group, the AUC presented a value of 0.925 (*p* < 0.001), with 100% of sensitivity and 79.3% of specificity, whereas in the moderate/severe endometriosis group the AUC was 0.868 (*p* < 0.001), with a sensitivity of 95.8% and specificity of 65.5%. These findings suggest that the expression of *BIRC5* may be a potential noninvasive biomarker for the diagnosis of endometriosis.

## 1. Introduction

Endometriosis is a common estrogen-dependent gynecological condition, which can affect women at a reproductive age [1]. It is defined as the presence of endometrial-like tissue outside of the uterus, often associated with chronic and inflammatory reaction [2]. The symptoms of endometriosis may vary from severe dysmenorrhea, dyspareunia, or chronic pelvic pain [1,2,3] to unexplained infertility, although the disease can be asymptomatic [4].

Cancer antigen 125 (CA125) plasma concentrations, a glycoprotein of epithelial origin, although largely used, are not reliable to diagnose endometriosis. Indeed, it may be elevated in several benign diseases and patients with non-ovarian malignancies, including cancers of the endometrium, lung, breast, pancreas, and colon [5], and it has no value in the diagnosis as a single test [3]. Indeed, according to guidelines of the European Society of Human Reproduction and Embryology (ESHRE), clinicians are recommended not to use immunological biomarkers, including CA125, in plasma, urine, or serum to diagnose endometriosis [6]. Despite considerable efforts towards searching for noninvasive diagnostic methods to detect endometriosis, the diagnosis can be suspected by ultrasound and/or other imaging methods [7] and confirmed only through laparoscopy with inspection of the abdominal cavity and histological confirmation of the lesion(s) [1,3]. As the surgery presents risks and also a high cost, a less invasive, but accurate test could lead to the diagnosis of the disease without the need for surgery, or at least it could help reduce the need for a surgical procedure for its confirmation [3].

The pathogenesis of endometriosis is still debated, although genetics, epigenetics, and immune elements may all play a pivotal role [8,9,10]. There are several theories to account for the origin of endometriosis and to explain how ectopic tissue can implant throughout the abdominal cavity [11]. However, there is no single theory that explains all of the different clinical presentations and pathological features in endometriosis [10]. A growing body of evidence has identified several comorbidities that are associated with endometriosis, including congenital uterine anomalies, autoimmune disease, allergy, cancers, and cardiovascular disease [12,13]. Melin et al. [14], based on 64,492 registers of the National Swedish Inpatient and Cancer Registrar data from 1969 to 2000, observed that women with endometriosis have an increased risk of some malignancies, particularly ovarian cancer. In addition, Wang et al. [15] in a recent meta-analysis comprising a total of 40,609 cases of epithelial ovarian cancer and 368,452 controls from 38 publications, also found that endometriosis was associated with an increased risk of epithelial ovarian cancer (OR = 1.42, 95% CI = 1.28–1.57). Endometriosis and cancer present similarities [16], such as cell invasion, unrestrained growth, neo-angiogenesis, and decreased apoptosis [17,18], although the first condition is clearly not neoplastic. 

Inhibitors of apoptosis proteins (IAPs) have emerged as modulators in an evolutionarily conserved step in apoptosis, as negative regulatory proteins that prevent apoptotic cell death. Survivin, a member of the IAP family, is encoded by the *BIRC5* (baculoviral IAP repeat-containing 5) gene located at 17q25, and it plays a role in cell division by inhibiting apoptosis and regulating the process of mitosis in embryonic cells during embryogenesis and in cancer cells during tumorigenesis and tumor metastasis [19]. It also participates in chromosome division and segregation, proliferation, stress response, and angiogenesis [20]. In addition, survivin is considered a key element for the metastasis phenomenon [21,22,23,24] and, consequently, has received significant attention as a potential oncotherapeutic target [25].

Survivin expression in normal endometrium shows cyclic alterations dependent on the menstrual cycle [26,27,28]. In addition, survivin overexpression is shown to be present in hormone-dependent endometrial disorders, such as endometrial hyperplasia, carcinomas, and endometriosis [27,28,29,30]. Therefore, the aim of the present study was to evaluate the expression of *BIRC5* in samples of peripheral blood of women with and without endometriosis.

## 2. Materials and Methods

### 2.1. Participants

This case-control study was performed between February 2017 and December 2018 and comprised 50 women recruited at the Human Reproduction and Genetics Center of the Faculdade de Medicina do ABC, Santo Andre, Brazil. The design, analysis, interpretation of data, drafting, and revisions followed the Helsinki Declaration and the strengthening the reporting of observational studies in epidemiology (STROBE) statement, available through the enhancing the quality and transparency of health research (EQUATOR) network (www.equator-network.org). The study design was approved by the independent Research Ethics Committee of the “Faculdade de Medicina do ABC” (approve code CAEE 64005816.8.0000.0082, approved on 1 February 2017). Each patient enrolled in this study signed an informed consent for all the procedures and to allow data and biological sample collection and analysis for research purposes. The study was non-advertised, and no remuneration was offered to encourage patients to give consent for the collection and analysis of their data. An independent data monitoring committee evaluated the interim and final data analysis of the study.

The endometriosis group comprised 40 women who had endometriosis diagnosed by laparoscopy and histological confirmation, classified according to the revised American Society for Reproductive Medicine (rASRM) score [31]. In this group, minimal/mild (stage I and II) endometriosis was found in 33.3% (12/36) of the cases, whereas moderate/severe (stage III and IV) endometriosis was found in 66.7% (24/36) of the cases. The surgical indication for all patients was infertility. The control group was carefully selected and comprised of 10 healthy and non-menopausal women who had no personal and/or familial history of endometriosis, autoimmune diseases, or cancer. All these women previously underwent tubal ligation for family planning reasons, and the absence of endometriosis was confirmed through inspection of the pelvic cavity. 

### 2.2. Sample Collection

Fifteen milliliters of the peripheral blood samples were collected in a tube containing clot-separator gel and in a tube containing PAXgene Blood RNA (PreAnalytiX, BD Diagnostics^®^, Franklin Lakes, NJ, USA). After collection, the tubes for the biochemical dosages were centrifuged (1000 rpm for 10 min), the plasma was aliquoted into microtubes and frozen at −80 °C for further determination of follicle-stimulating hormone (FSH), luteinizing hormone (LH), progesterone, prolactin, and CA125 concentrations. The tube for RNA extraction was stored at −80 °C until extraction.

The samples for RNA extraction were collected in the three phases of the menstrual cycle (follicular, ovulatory, and luteal) for all women of the control group. Among the women of the endometriosis group, the samples were collected in 38.9% of women (14/36) in the follicular phase, 27.8% (10/36) in the ovulatory phase, and in 33.3% (12/36) in the luteal phase.

### 2.3. Hormonal Measurement

The hormonal profile was measured during the investigation into the cause of infertility. Progesterone and prolactin were measured at the luteal phase and FSH and LH at the follicular phase of the menstrual cycle. The hormones were measured by enzyme-linked fluorescent immunoassay (BioMerieux^®^, Hazelwood, MO, USA).

### 2.4. RT-qPCR

RNA extraction was carried out with Qiazol Lysis Reagent according to the manufacturer’s instructions (Qiagen Inc., Valencia, CA, USA) and then total RNA was treated with DNase-I endonuclease (Thermo Fisher Scientific, Waltham, MA, USA). RNA sample concentrations were analyzed using a Nanodrop 2000 spectrophotometer (Thermo Fisher Scientific, Waltham, MA, USA) and the RNA integrity was analyzed via agarose gel electrophoresis to identify the 28S and 18S ribosomal rRNA. The cDNA synthesis was done with 1 µg of total RNA using a high capacity cDNA reverse transcription kit (Thermo Fisher Scientific, Waltham, MA, USA) following the manufacturer’s guidelines.

The expression of *BIRC5* (Hs04194392_s1) and glyceraldehyde3-phosphate dehydrogenase (GAPDH, Hs99999905_m1) genes was measured by RT-qPCR, based on the TaqMan methodology (ThermoFisher Scientific, Waltham, MA, USA) using the equipment StepOne Real-Time PCR System (Applied Biosystems, Foster City, CA, USA). PCR reactions were processed to a final volume of 20 mL containing 10 μL of 2× TaqMan Universal PCR Master Mix, 1.25 μL TaqMan assay (20×), 2 μL of sample cDNA, and 6.75 μL of RNAse-free water. Reactions were performed at 95 °C for 10 min, followed by 40 cycles of 95 °C for 15 s, and annealing/extension at 60 °C for 60 s. Each reaction was performed in triplicates. The gene expression results were obtained using the 2^−ΔCt^.

### 2.5. Statistical Analyses

Statistical analyses were performed using GraphPad Software (v.7, LLC, San Diego, CA, USA, https://www.graphpad.com). Data normality was verified with the Shapiro–Wilk test. Variables were presented by medians with 95% confidence intervals (CI). Differences between two groups were tested by Mann–Whitney or Kruskal–Wallis tests. A Spearman’s correlation test was performed to analyze the correlation between hormonal levels and the *BIRC5* expression. To test for accuracy, receiver operator characteristic (ROC) analysis was used and specificity, sensibility, predictive values, and 95% confidence interval (95% CI) were calculated. Statistical significance was considered when *p* < 0.05.

## 3. Results

Table 1 shows the comparison of clinical and hormonal characteristics of women with and without endometriosis. Hormonal values were in accordance with the reference values for each phase of the menstrual cycle. CA125, FSH, and prolactin levels were significantly higher in women with endometriosis compared with the control group. Conversely, age, body mass index (BMI), LH, and progesterone levels were not significantly different between groups. Regarding the endometriosis stage, all the parameters were not significantly different between women with minimal/mild and moderate/severe disease.

**Figure 1:**
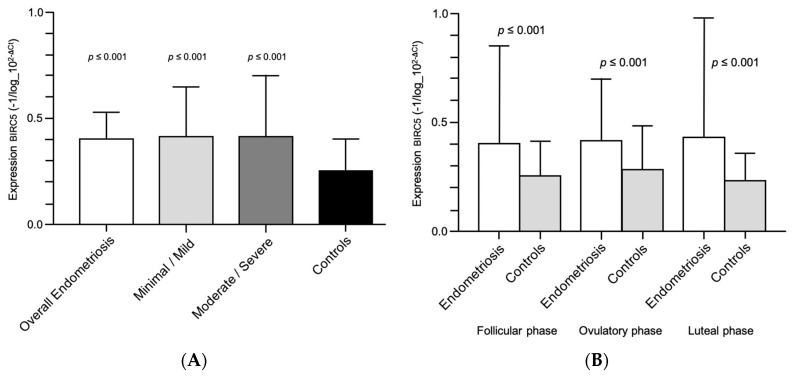
(**A**) *BIRC5* expression in the peripheral blood samples of women with endometriosis according to the disease stage. (**B**) *BIRC5* expression in the peripheral blood samples of women with and without endometriosis in different phases of the menstrual cycle.

Figure 1A shows the comparison of *BIRC5* expression among women with endometriosis and according to the disease stage and the control group. The *BIRC5* expression was also significantly higher in women with endometriosis, regardless of the endometriosis stage (minimal/mild and moderate/severe endometriosis). Figure 1B shows the comparison of *BIRC5* expression between women with and without endometriosis in different phases of the menstrual cycle. The *BIRC5* expression was significantly higher in all phases of the menstrual cycle in women with endometriosis. 

The correlation between hormonal levels and *BIRC5* expression in peripheral blood of women with endometriosis is reported in Table 2. Spearman’s correlation coefficient showed that the progesterone was correlated with *BIRC5* expression (rho = 0.382, *p* = 0.045).

**Figure 2:**
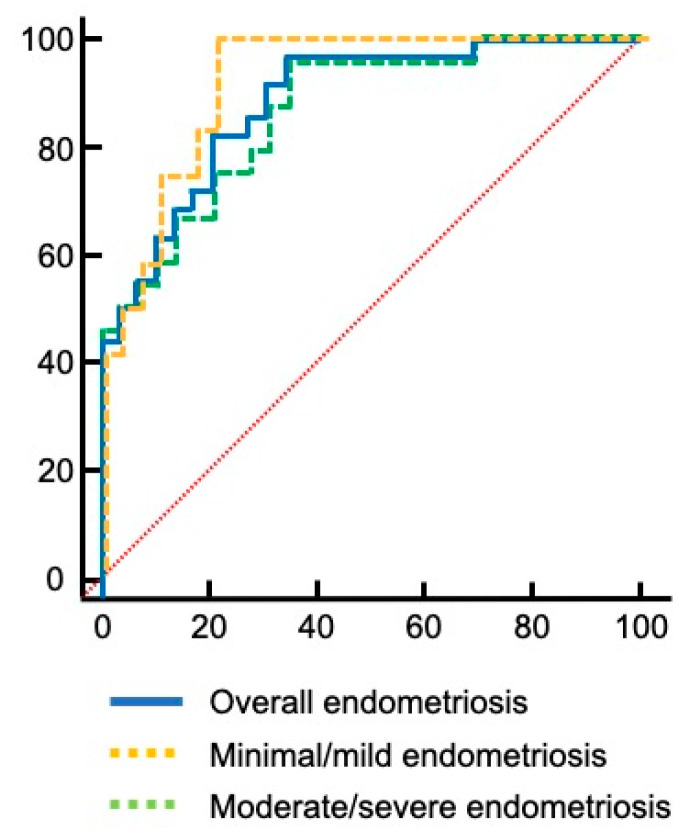
The accuracy of *BIRC5* expression in the peripheral blood for the diagnosis of endometriosis. Red dotted line indicates line of no-discrimination.

To estimate the accuracy of *BIRC5* expression in the peripheral blood to diagnostic endometriosis and also according to disease staging, the area under the ROC curve was analyzed (Figure 2). 

Considering the overall endometriosis group, the area under the curve (AUC) was 0.887 (95% CI = 0.809–0.965; *p* < 0.001), with a cut-off value of 2^−ΔCt^ > 0.00030, 97.2% of sensitivity and specificity of 65.5%; with a positive predictive value of 68.5% (95% CI 61.0–75.2) and a negative predictive value of 92.8% (95% CI 64.2–98.9). Regarding the minimal/mild endometriosis group, the AUC presented a value of 0.925 (95% CI = 0.848–1.0; *p* < 0.001), with a cut-off value of 2^−ΔCt^ > 0.0018, sensitivity of 100% and specificity of 79.3%, positive predictive value 66.7% (95% CI 49.5–80.3), and negative predictive value 100%. For the moderate/severe endometriosis group, the AUC was 0.868 (95% CI = 0.775–0.962; *p* < 0.001), with a cut-off value of 2^−ΔCt^ > 0.00030, sensitivity of 95.8% and specificity of 65.5%, with a positive predictive value of 69.7% (95% CI 58.0–79.3) and a negative predictive value of 95.0% (95% CI 73.3–99.2).

## 4. Discussion

In the current study, the expression pattern of *BIRC5* as a potential non-invasive biomarker was assessed in the peripheral blood samples taken during different phases of the menstrual cycle of women with and without endometriosis. Our results showed that *BIRC5* is differently expressed in women with endometriosis compared with healthy controls, regardless of the endometriosis stage.

Some findings have highlighted the main role of inflammation in endometriosis by acting on proliferation, apoptosis, and angiogenesis. Nevertheless, the mechanisms underlying this disease are still unclear [32]. Homeostasis maintenance of tissue is mainly regulated by cell death and some studies have shown that apoptosis increases during the menstrual cycle to retain cell homeostasis, removing aged cells from the functional layer of the endometrium [33]. The rate of apoptosis is decreased in endometrial cells of women with endometriosis, and therefore, it may contribute to the pathogenesis of the disease [34,35,36,37,38].

In apoptosis inhibition, survivin has a key role in both intrinsic and extrinsic pathways of apoptosis [39,40,41,42,43,44]. Considering the aspects of an immune response, survivin modulates the apoptotic threshold of neutrophils and its expression increases during inflammatory reactions in these cells. Survivin has also a contribution to T-cell development, maturation, activation, and homeostasis [20,42]. Its expression increases after the activation of naive T cells in lymphoid organs, showing the importance of survivin in the initiation of immune responses. The increased level of survivin has been documented in serum and lymphocytes of patients with different autoimmune diseases [20,42,43]. Numerous studies have shown that peritoneal leukocytes and their inflammatory mediators exert local effects, creating a microenvironment that may facilitate the development and progression of endometriotic lesions. Besides, some authors have suggested that endometriosis may have, at least in part, an autoimmune component [43,44].

Zwerts et al. [45] observed that the structures of the embryo show high expression of survivin, while the absence of its expression in endothelial cells contributes to the death of the embryo. Other studies also demonstrated that the presence of survivin is essential for normal development and organogenesis. Survivin’s involvement in the regulation of endothelial cell survival and its influence in maintaining vascular integrity has paramount importance in neurogenesis, angiogenesis, and cardiogenesis. The survival of undifferentiated pluripotent stem cells is highly dependent on anti-apoptotic factors, such as survivin [46,47], and overexpression of survivin in embryonic stem cells, pluripotent cells and somatic stem cells [48,49], as well as the correlation between higher *BIRC5* expression and lower cell differentiation in cells derived from bone marrow [47] is probably due to the fact that bone marrow is a source of hematopoietic stem cells and mesenchymal stem cells [50,51].

All these findings corroborate different theories for the origin of endometriosis, such as the theory of endometrial stem cells [52] or the increase in transient progenitor cells in which circulating stem cells originating from bone marrow or the basal layer of the endometrium can differentiate into endometrial tissue in different anatomical locations; or the theory of genetic/epigenetic changes in which, regardless of the origin of the initial cell, genic variants or epigenetic changes associated with changes in the peritoneal environment, such as inflammatory, immunological and oxidative stress, could initiate diseases in their different forms (ovarian, peritoneal, deep, and lesions outside the pelvis) and thus explain its complexity [11,53], which may lead also to significant anatomical alterations and make the surgical approach difficult [54]. Recently, a systematic review that summarized the findings from 21 studies and 1263 women with endometriosis reported that survivin (gene and/or protein) expression is increased in endometriosis, regardless of the methodology used (real-time reverse transcription polymerase chain reaction (RT-PCR), quantitative PCR (RT-qPCR), immunohistochemistry, Western blot, or enzyme-linked immunosorbent assay (ELISA)), sample studied (endometrium or blood), the phenotype of the endometriosis (superficial, ovarian, and deep) or morphology of the endometriotic lesions (pigmented or non-pigmented) [43].

Zang et al. [28] observed that the presence of paracrine factors produced by normal endometrial stromal cells mediated the effect of progesterone on glandular endometriotic cells in vitro. The authors also found that endometriotic stromal cells have lost the ability to regulate apoptotic signaling in endometriotic gland cells that grow in ectopic sites, while these cells have not lost their ability to respond to paracrine factors produced by endometrial stromal cells. The observation of the cyclic expression of survivin in normal endometrial cells suggests that the expression of the *BIRC5* gene is influenced by steroid hormones and deregulated by the increase in progesterone in the luteal phase. Progesterone is a potent antagonist of estrogen-induced endometrial proliferation and plays an important role in the pathogenesis of endometriosis [55]. The continuous use of progestogens, as well as the combined use of estrogens and progestogens in the treatment of endometriosis results in the inhibition of endometrial growth, with consequent atrophy of the lesions, in addition to being associated with anti-inflammatory action, suppression of metalloproteinases, and inhibition of angiogenesis [56]. In the present study, Spearman’s correlation showed that progesterone level was correlated with *BIRC5* expression (rho = 0.382, *p* = 0.045).

Acimovic et al. [57] studied survivin expression in 30 women with endometriosis and 10 women without the disease. The authors found a difference in the expression of survivin in peripheral blood between the groups (*p* = 0.025) and the results demonstrated that the accuracy of survivin as a diagnostic test for endometriosis was 70%, with a sensitivity of 66.7% and specificity of 80%. However, the study does not report the phase of the menstrual cycle during which the samples were collected. In our study, the expression of *BIRC5* in the peripheral blood of women with endometriosis showed an accuracy of 88.7%, with a sensitivity of 97.2% and specificity of 65.5%. In minimum/mild endometriosis the accuracy was 92.5%, with 100% sensitivity and 79.3% specificity, whereas in moderate/severe disease the accuracy was 86.8% with 95.8% sensitivity and specificity of 65.5%. The data suggest that *BIRC5* expression may be a potential minimally invasive biomarker in the diagnosis of endometriosis.

Some studies suggest that prolactin may also act as a probable prognostic biomarker to differentiate patients with endometriosis according to the stage of the disease and also as an indicator of endometriosis related-infertility since higher levels are observed in women with endometriosis when compared with infertile women without endometriosis; however, this relation is debatable [58]. Prolactin plays an important role in the immune system, participating in the inflammatory process, angiogenesis, and in the formation of thrombi and scarring [59]. In our study, we observed that women with endometriosis had higher levels of prolactin, despite being within the reference value, when compared to fertile women without the disease; in addition, we did not find a significant difference for prolactin values considering the stage of the disease (13.5 ng/mL (7.6–19.3) versus 15.2 ng/mL (10.5–20.2), respectively in minimal/mild and moderate/severe endometriosis; *p* = 0.410). Nonetheless, minimal/mild disease was found in only one-third of the women enrolled in this study. Indeed, 66.7% of the women in the endometriosis group were classified as advanced (III/IV) stages according to the rASRM, and this could be considered in line with the enrollment of women with endometriosis-associated infertility.

For the correct interpretation of our findings, some limitations of the present study should be taken into account. We studied infertile women with endometriosis and fertile women without the disease, and the mechanisms responsible for the association of infertility and endometriosis are still not fully elucidated. As women with endometriosis were undergoing assisted reproduction treatment and the strict inclusion and exclusion criteria of the study participants, we were unable to obtain samples from the same participant at different phases of the menstrual cycle.

## 5. Conclusions

In conclusion, increased expression of the *BIRC5* gene in the peripheral blood of women with endometriosis may indicate their role in cell proliferation and anti-apoptotic activity in the development of the disease. The findings suggest that the expression of *BIRC5* may be a potential noninvasive biomarker for the diagnosis of endometriosis. 

Increased knowledge of the pathophysiologic mechanisms of endometriosis is crucial for an early and accurate diagnosis, which can reduce the costs associated with the management of the disease and help to avoid (or at least reduce) the negative impact on the physical and psychosocial health of the patients.

More studies however are needed to confirm the applicability of the proposed biomarker of endometriosis for clinical use.

## Figures and Tables

**Table 1:** Comparison of clinical and hormonal characteristics of women with and without endometriosis.

Variable *	Endometriosis (*n* = 36)	Controls (*n* = 10)	*p* **
Age (years)	35 (33.0–38.0)	33 (32–34.5)	0.933
BMI (kg/m^2^)	24.3 (23.1–25.4)	24.7 (23.8–25.7)	0.800
CA125 (mUI/mL)	49.8 (22.6–67.6)	18.9 (15.2–36.3)	<0.001
FSH (mUI/mL)	7.2 (6.8–8.2)	6.4 (6.1–6.9)	<0.001
LH (mUI/mL)	6.3 (4.3–8.3)	6.7 (5.0–8.3)	0.838
Progesterone (ng/mL)	8.9 (6.9–11.0)	5.9 (2.9–8.9)	0.061
Prolactin (ng/mL)	17.1 (11.9–22.6)	8.5 (6.5–15.1)	0.010

* Median and 95% confidence interval. BMI, body mass index; CA125, cancer antigen 125; FSH, follicle-stimulating hormone; LH, luteinizing hormone. ** Mann–Whitney test.

**Table 2:** Correlation between hormone levels and *BICR5* expression in peripheral blood of women with endometriosis.

	rho *	*p*
CA125	−0.191	0.265
FSH	0.276	0.115
LH	0.274	0.117
Progesterone	0.382	0.045
Prolactin	−0.030	0.873

* Spearman’s correlation. CA125, cancer antigen 125; FSH, follicle-stimulating hormone; LH, luteinizing hormone.
